# Genetic Breeding and Diversity of the Genus *Passiflora*: Progress and Perspectives in Molecular and Genetic Studies

**DOI:** 10.3390/ijms150814122

**Published:** 2014-08-14

**Authors:** Carlos Bernard M. Cerqueira-Silva, Onildo N. Jesus, Elisa S. L. Santos, Ronan X. Corrêa, Anete P. Souza

**Affiliations:** 1 Laboratory of Applied Molecular Genetics, Department of Exact and Natural Sciences, State University of Southwest Bahia, Itapetinga 45700-000, Brazil; E-Mails: csilva@uesb.edu.br (C.B.M.C.-S.); elisalisboa@yahoo.com.br (E.S.L.S.); 2 Molecular Biology and Genetic Engineering Center, University of Campinas, CP 6010 Campinas, Campinas 13083-875, Brazil; 3 Brazilian Agricultural Research Corporation, Cassava & Fruits, Cruz das Almas 44380-000, Brazil; E-Mail: onildo.nunes@embrapa.br; 4 Biotechnology and Genetic Center, Biological Sciences Department, State University of Santa Cruz, Ilhéus 45662-900, Brazil; E-Mail: ronanxc@uesc.br; 5 Plant Biology Department, Biology Institute, University of Campinas, Campinas 13083-875, Brazil

**Keywords:** active germplasm banks, genetic diversity, genetic engineering, molecular marker, passion fruit breeding, population genetics

## Abstract

Despite the ecological and economic importance of passion fruit (*Passiflora* spp.), molecular markers have only recently been utilized in genetic studies of this genus. In addition, both basic genetic researches related to population studies and pre-breeding programs of passion fruit remain scarce for most *Passiflora* species. Considering the number of *Passiflora* species and the increasing use of these species as a resource for ornamental, medicinal, and food purposes, the aims of this review are the following: (i) to present the current condition of the passion fruit crop; (ii) to quantify the applications and effects of using molecular markers in studies of *Passiflora*; (iii) to present the contributions of genetic engineering for passion fruit culture; and (iv) to discuss the progress and perspectives of this research. Thus, the present review aims to summarize and discuss the relationship between historical and current progress on the culture, breeding, and molecular genetics of passion fruit.

## 1. Introduction

### 1.1. General Characteristics of the Genus Passiflora, Family Passifloraceae

The family *Passifloraceae Juss*. ex DC. belongs to the order Violales, class Magnoliopsida, and phylum Magnoliophyta. Although records of plants that belong to this family have been in existence since 1553, there have been disagreements regarding the number of genera and species in the family since the first literary record of a plant in this group was written in Colombia [1]. Over the past few centuries, several scientific studies have been published describing the species and the organization of the species into genera and subgenera. Estimates of the number of species in the *Passifloraceae* family vary between 700 [2] and 520 [3,4,5]. Such variations are the result of taxonomical uncertainties, the use of synonyms, and descriptions of new species. There is also no consistency in the number of genera, which varies between 18 [2,3] and 23 [6].

Despite taxonomical uncertainties, *Passiflora* is highly diverse, with approximately 520 species [5]. Approximately 96% of the species are distributed in the Americas, although there are records of species in India, China, Southeastern Asia, Australia, the Pacific islands, and neighboring regions (examples of species include *Passiflora aurantia*, *Passiflora cinnabarina*, *Passiflora herbertiana*, *Passiflora cupiformis*, *Passiflora henryi*, *Passiflora jugorum*, *Passiflora moluccana*, and *Passiflora siamica*). Brazil and Colombia, in particular, are centers of diversity; approximately 30% of *Passiflora* species are found in these countries (approximately 150 in Brazil and 170 in Colombia), including 89 that are endemic to Brazil [7,8,9,10].

Based on the progress in taxonomic and phylogenetic studies using molecular markers and sequencing techniques, particularly over the last 10 years, the taxonomic classification of *Passiflora* species has been organized into four subgenera: *Astrophaea*, *Decaloba*, *Deidamiodes*, and *Passiflora* [11,12]. Although this infrageneric organization into only four subgenera diverges from the hypothesized 22 or 23 subgenera [13,14], research based on phylogenetic approaches, morphometric data, genotypic data of microsatellite markers [15] and DNA fragments from seven regions of the passion fruit genome (the *rbcL* and *rps4* genes, the *trnL* intron and *trnL-F* intergenic spacers from the plastid genome, the *nad1* b/c and *nad5* d/e introns from the mitochondrial genome, and a portion of the *26S* gene from the nuclear ribosomal genome) [16] supports this simpler division of the genus. Together, the information available for passion fruit supports the infrageneric classification of *Passiflora* based on studies of molecular phylogeny that are corroborated by morphological and ecological characteristics [16,17,18].

**Figure 1:**
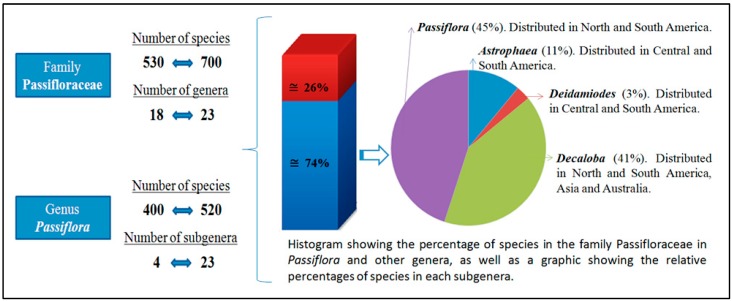
Schematic illustrating general information regarding the *Passifloraceae* family (the number of genera, species, and distribution within different taxonomic levels) and details of the occurrence of *Passiflora* in Brazil. The data presented were summarized from the results of previous studies [2,3,4,9,12].

Figure 1 presents a summary of the data discussed above. Additional information on the family Passifloraceae, the group’s taxonomy, and the occurrence of species is reported in the List of Species of Brazilian Flora [10] and in the book *Passiflora: Passionflowers of the world* [19].

Molecular studies of passion fruit based on the combined and individual analysis of DNA sequences from the nuclear, plastid, and mitochondrial genomes also contribute directly to (i) estimates of the origin of the genus *Passiflora* and the diversification of its subgenera obtained through the analysis of molecular dating [16]; (ii) the identification and understanding of different organellar inheritance patterns [20]; and (iii) the demonstration of the potential use of fragments from the internal transcribed spacer (ITS) regions of nuclear ribosomal sequences and variations in the plastidial sequences to study inter- and intraspecific variability in *Passiflora* species [21,22]. Furthermore, the contributions to molecular phylogeny may be observed in in-depth studies [16,17,18].

With the expansion of the genomic information (sequences) available in public databases as well as the availability of genetic material in collections, germplasm banks, and herbaria, the use of short orthologous DNA sequences (DNA barcodes) to identify species can contribute considerably to the characterization and utilization of biological diversity [23,24]. In particular, for groups such as the genus *Passiflora*, for which taxonomic uncertainties exist and new species are continuously being discovered (approximately five species per year) [25], the definition and use of DNA barcodes can contribute directly to germplasm characterization and the evaluation of diversity in natural populations.

The inherent variability of *Passiflora* is not restricted to its morphological characteristics [26,27] and the physical and chemical compositions of the fruit and flowers [28,29,30,31] but includes traits important for its survival in natural environments and/or cultivation conditions, such as the resistance of wild species to biotic (fungi, bacteria, nematodes, and viruses) and abiotic stresses (variations in the edaphoclimatic characteristics of macro- and microregions in which *Passiflora* species naturally occur or are cultivated) [32,33,34]. Such variability may be useful for breeding programs with different end results.

Results from cytogenetic research on the genus *Passiflora* are scarce and restricted to less than 20% of the species [35]. Although such studies are increasing [36,37], this area of knowledge remains open to basic investigation. Despite this scarcity of cytogenetic information, research has contributed to our understanding of the group’s evolutionary relationships and has facilitated direct interspecies crosses and thus the genetic breeding of passion fruit. Certain species, particularly those of economic interest (due to fruit production and ornamentation, such as *Passiflora* *edulis* Sims, *Passiflora alata* Curtis, *Passiflora coccinea* Aubl, and *Passiflora incarnate* L.), are described by cytogenetic information available at different levels, including data from molecular cytogenetic techniques, such as fluorescent *in situ* hybridization (FISH) and genomic *in situ* hybridization (GISH) [38,39,40,41]. Details outlining the use of cytogenetic techniques in *Passiflora* are available [36,37]. Molecular data were also recently made available from the construction and initial characterization of a genomic library of *P. edulis* using the bacterial artificial chromosome (BAC) technique [42,43], which should contribute to the characterization of the germplasm, construction of chromosome maps of *P. edulis* and neighboring species, and identification of interspecies hybrids.

In relation to estimates related to the genome size of passion fruit species, as is discussed in the review presented by Souza *et al.* [37], the studies are very limited, and for most species, the information was derived from works carried out in the last 10 years with use of flow cytometry. This methodology may contribute to the estimation of DNA content as an aid in the analysis of ploidy, the identification of hybrids and the establishment of correlations between the genome sizes and biological and agronomic characteristics [37,44]. The few studies investigating the genome sizes of passion fruit have indicated significant interspecific variation, such as the results reported by Souza *et al.* [37] for eight species (with 2C nuclear DNA content ranging from 1.83 pg in *Passiflora suberosa* to 5.36 pg in *Passiflora quadrangularis*) and the more recent results from Yotoko *et al.* [44] for 50 species (indicating variation of 2C content from 0.42 pg *in P. organogenesis* to 4.41 pg in *P. alata*).

The available cytogenetic information on passion fruit species describes four different chromosome numbers distributed among the four subgenera of *Passiflora*: *x* = 6 in *Decaloba*, *x* = 9 and *x* = 10 or 11 in *Passiflora*, *x* = 10 in *Astrophaea*, and *x* = 12 in *Deidamiodes* [5,39]. This distribution is largely in accordance with the infrageneric classification supported by molecular phylogeny for the genus *Passiflora*. Although there is no precise estimation of the basic number of chromosomes across the genus, studies have suggested 6 or 12 as the precise number [35,38]. In the case of polyploidy, most *Passiflora* species are diploid, with 2*n* = 12, 18, or 20 chromosomes. *Tetra-*, *hexa-*, or *octoploid* species are rare [36,39]. The most commercially important species is diploid (2*n* = 18), which favors genetic breeding to obtain interspecies hybrids [36,43,44].

### 1.2. Economic Importance of Passion Fruit Species

**Figure 2:**
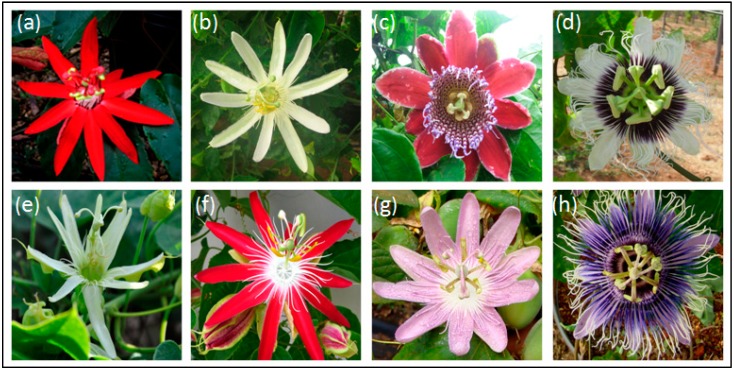
Panel illustrating flowers from passion fruit species of ornamental ((**a**) *Passiflora coccinea*; (**b**) *Passiflora* *mucronata*; (**c**) *Passiflora* *alata*) as well as examples of ornamental passion fruit hybrids ((**d**)BRS Rubi do Cerrado; (**e**)BRS Pérola do Cerrado; (**f**) BRS Estrela do Cerrado; (**g**)BRS Rosea Púrpura; (**h**)BRS Céu do Cerrado). Credits: F.G. Faleiro & NTV Junqueira, researchers from the Embrapa Cerrado, Brazil.

Several segments of society display a great interest in passion fruit plants as ornamental and medicinal plants, as a source of oils for the cosmetic industry, and for their fruits and fruit derivatives. The use of passion fruit as an ornamental plant is justified because of the diversity and beauty of its leaves, flowers, and fruit, and more than 400 ornamental hybrids exist worldwide [45] (Figure 2). However, the use of passion fruit as an ornamental plant has only recently begun in Brazil, as the first hybrids for ornamental use were developed and launched in the last decade: the “Brasil (BRS) Estrela do Serrado” (N.Ref. RNC-MAPA 21717) was obtained from a cross between *P. coccinea* and *Passiflora setacea*, “BRS Rubiflora” (N.Ref. RNC-MAPA 21718) was obtained from a cross between the F_1_ hybrid (*P. coccinea* and *P. setacea*) with *P. coccinea*, and “BRS Roseflora” (N.Ref. RNC-MAPA 21715) was obtained from the cross between the F_1_ hybrid (*P. coccinea* and *P. setacea*) with *P. setacea*. These ornamental hybrids were developed at Embrapa Cerrados, Planaltina, Brazil (images and details are available at [46]). Ornamental hybrids have also been produced in Brazil’s northeast region, including “*Passiflora* *alva*”, “*Passiflora* *priscilla*” and “*Passiflora* *aninha*” all of which were obtained from a cross between *Passiflora* *palmeri* and *Passiflora* *foetida*. These ornamental hybrids were developed at the Universidade Estadual de Santa Cruz, Ilhéus, Bahia, Brazil [47] (images and details are available at [48]).

The identification of species with morphological and adaptive characteristics for ornamental use has increased in Brazil, particularly in research centers, such as the Universidade Estadual de Santa Cruz, Ilhéus, BA, Brazil; Embrapa Cerrados, Embrapa Mandioca e Fruticultura, Cruz das Almas, BA, Brazil; and the Instituto Agronômico, Campinas, São Paulo, Brazil. Among the various passion fruit species with ornamental potential, the characterization and utility of ornamental hybrid generation of the following species is distinctive, at least at the institutions noted above: *P. coccinea*, *P. foetida*, *Passiflora* *morifolia*, *Passiflora mucronata*, *P. palmeri*, *P. setacea*, *P. suberosa*. For readers interested in a deeper understanding of the subject, we suggest reading the specific research of Abreu *et al.* [49] and Costa *et al.* [50].

The use of *Passiflora* species as medicinal plants has been previously documented. The leaves, flowers, roots, and fruit of wild and commercial species are known to fight diseases, such as helminth infestations, gastric tumors, and stress, and are an integral component of the cultural tradition of many populations [50]. Passion fruit is a rich source of minerals and Vitamins A, C, and D [51] as well as a source of alkaloids, flavonoids, and carotenoids that are beneficial to human health [52]. Passion fruit seeds are sources of essential fatty acids (55%–66% linoleic acid, 18%–20% oleic acid, and 10%–14% palmitic acid), which may be used in the food and cosmetic industries [53]. The potential commercial use of passion fruit also includes the extraction of oils for the manufacturing of soaps, creams, shampoos, and other products by the cosmetic industry [54]. Compounds in passion fruit plants with anxiolytic, antihypertensive, sedative, and analgesic properties are well known [55,56]. Considering the research presented above, the following species stand out among the various passion fruit species with potential medicinal properties: *P. alata*, *Passiflora* *caerulea*, *P. edulis*, *P. foetida*, *Passiflora* *incarnata*, *Passiflora* *laurifolia*, and *Passiflora* *maliformis*.

Despite the ecological importance and different potential uses of *Passiflora* as a genetic resource, it is the production of fruit for food and commercially produced juice that justifies the cultivation of the passion fruit plant [57] and that has led to increasing academic and economic interest. Brazil is the largest producer and consumer of passion fruit [58,59]. National production reached approximately 776,000 tons in 2012 [60]. Although the cultivation of passion fruit occurs in all regions of the country, northeastern Brazil produces approximately 73% of the national product [60]. Approximately 90% of the area designated for passion fruit culture is cultivated with *P. edulis*, and *P. alata* is the second most cultivated species of the genus [54,61]. Although they represent a small proportion of national production, the cultivation and consumption of fruits from wild species, such as *Passiflora cincinnata*, *Passiflora* *nitida*, *P. quadrangularis*, and *P. setacea*, have been reported in the literature [31,57,61,62,63].

Brazil’s position as the largest passion fruit producer is due to the large size of the area cultivated rather than its productivity (Table 1). Data from the Brazilian Institute of Geography and Statistics, shown in Table 1, indicate an increase in the area devoted to passion fruit cultivation and fruit production but do not indicate a corresponding increase in productivity. The change in productivity of passion fruit cultivation was no more than 4% over the last 10 years despite the available natural diversity and progress in genetic breeding techniques. Furthermore, the recent mean productivity (14 tha^−1^) in Brazil (Table 1) is less than 30% of the potential productivity rate estimated for passion fruit cultivation (50 tha^−1^·year^−1^) with the use of improved cultivars and techniques [61].

The major factors that limit productivity are the absence of productive varieties that are adapted to local conditions, a lack of resistance to major diseases, and the use of seeds obtained by open pollination from plants in the planted area, which generates low-quality fruit and inconsistent production [30,32,64]. These conditions primarily occur because the available improved genotype selections for producers are scarce [61,64,65]. The *Passiflora* genetic resource exhibits variability in resistance to major passion fruit diseases and a high morphological variability, which together may contribute to disease control or the diversification of products. Although the potential for wild and commercial species is remarkable, basic studies involving the agronomic characterization of the species must be initiated.

Because molecular biology in general and the use of molecular markers in particular provide a wide range of useful tools for basic and applied research associated with genetic breeding at different stages (from the prospect of germplasms to the characterizations required for pre-breeding and the protection of cultivars in the post-breeding period), success is expected for passion fruit breeding programs in the future. Therefore, the current review will address the available knowledge on this subject (with tables and diagrams to represent the data quantitatively and qualitatively) and discuss the relevant studies and resulting knowledge, although this review does not pretend to end the discussion on the current and potential use of molecular markers to characterize the genus *Passiflora* and for plant breeding.

**Table 1:** Descriptive presentation of the Brazilian agricultural production * of passion fruit between 2003 and 2012.

Period (Years)	Planted Area (*ha* × 1000)	Production (*t* × 1000)	Productivity (*t*/*ha*)
2003	34.9	485.3	13.9
2004	36.6	491.6	13.4
2005	35.8	479.8	13.4
2006	44.4	615.2	13.9
2007	46.8	664.3	14.2
2008	48.7	684.4	14.0
2009	50.8	718.8	14.2
2010	62.0	920.2	14.8
2011	61.6	923.0	14.9
2012	57.8	776.1	13.4
Average (CV)	48 (21%)	676 (24%)	14 (4%)

* Original data were obtained from the Municipal Agricultural Production presented by the Instituto Brasileiro de Geografia e Estatística [60] between 2003 and 2012; CV = coefficient of variation.

## 2. Contributions of Molecular and Genetic Studies for the Characterization and Use of *Passiflora* Biodiversity

### 2.1. Biodiversity, Conservation, and Breeding of Passion Fruit

**Figure 3:**
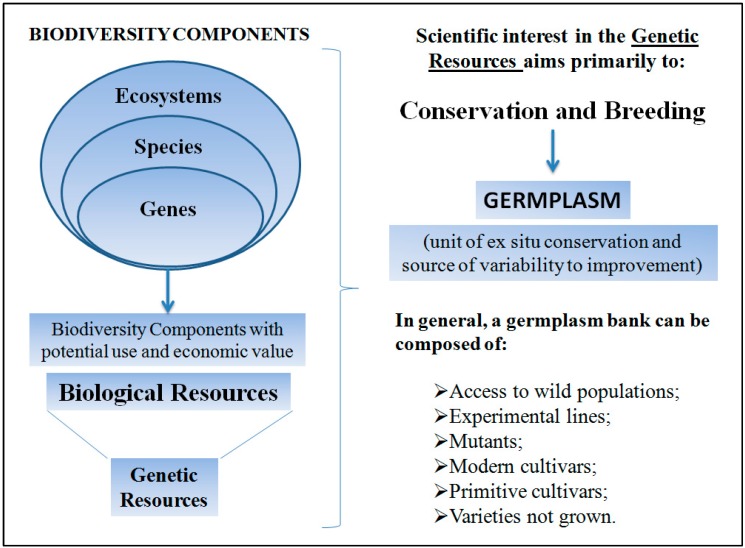
Schematic illustrating the different components of biodiversity and the classification of those components that have generated immediate interest or are of potential use for society, scientific interests, or conservation and breeding. The data presented were summarized from the results of previous work [66,67].

The concept of biodiversity may vary according to the context in which it is employed. Biodiversity may be defined as the totality of genes, species, and ecosystems of a specific region or the planet as a whole. This totality is the result of evolutionary processes [66,67]. The components of biodiversity with an immediate and/or potential interest for humans constitute a biological resource that may, in turn, be classified as a genetic resource [67] including the genetic variability of social and economic assets. In general, genetic resources are considered sources of variability (natural raw material) both for breeding programs and to support conservation strategies. However, several characterizations that involve different methodological approaches are required to use genetic resources for the conservation of biodiversity and genetic breeding. We have devised an illustrative scheme to facilitate the understanding and visualization of the relationships among the presented concepts (Figure 3). However, for readers interested in expanding on this theme, we also recommend the reading of specialized texts [68].

Although *Passiflora* has a high species diversity, and despite the dispersion of this genus within different tropical biomes, information on the intra- and extraspecies genetic diversity is rare for most species. Research available in the primary databases of scientific publications (Scopus [69] and Web of Science [70]) includes characterizations of genetic diversity based on molecular markers of fewer than 60 species (details are provided in Table 2 and discussed in the following sections). Nearly all these studies were performed using dominant markers, which do not allow for the maximum exploitation of genetic information because it is impossible to distinguish heterozygote genotypes, and were based on a few commercial genotypes with only local interest and/or a few wild accessions per species. Therefore, these studies cannot be characterized as population genetics studies.

**Figure 4:**
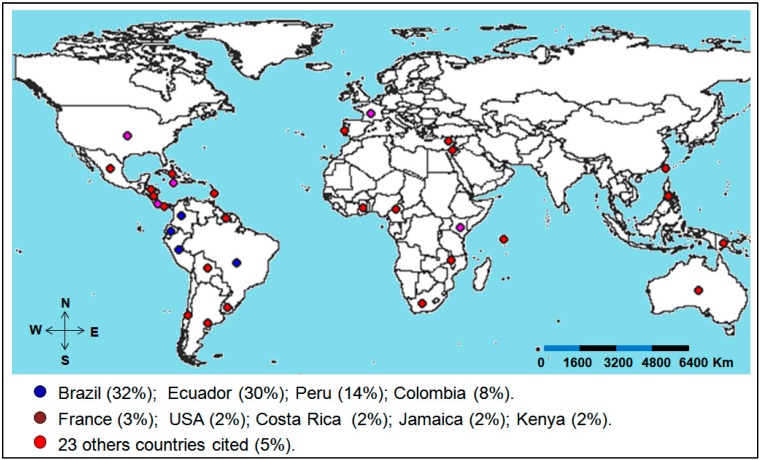
Map showing the countries where worldwide collections of passion fruit (*Passiflora* spp.) are located. The countries with the most representative collections are identified with blue circles (four countries, representing approximately 84% of accessions), followed by countries identified with magenta circles (five countries, representing approximately 11% of accessions) and countries identified with red circles (23 countries, representing approximately 5% of accessions). Note: The percentages of accessions were calculated from the data presented by Ferreira [59].

Similar to the lack of information available on the cytogenetics (discussed in the introduction of this review) and genetic diversity of *Passiflora*, the number of accessions present in collections and active germplasm banks (BAGs) of passion fruit are considered incipient in Brazil and across the world [27,54,59]. At least 1200 accessions of *Passiflora* spp. are estimated and are preserved in 50 collections of germplasms distributed in approximately 32 countries (Figure 4) [54,59]. Among these countries, we highlight Brazil, Ecuador, Peru and Colombia, which account for approximately 84% of accessions. Although it is likely that the exact number of accessions kept in these collections has varied since the compilations made by Ferreira [59], the predominance of accessions in South American collections present in tropical countries is expected because this region is considered as a center of diversity of the genus *Passiflora* [7,8,9,10]. In addition, these same countries are recognized as leading producers of passion fruit. [59]. Although Brazil is considered an important center of genus diversity, extant collections contain fewer than 70 species of the genus, or approximately 50% of the Brazilian *Passiflora* species and less than 15% of all species within the genus [59]. Furthermore, the majority of accessions in BAGs lack biological, genetic, and molecular characterizations. This dearth of information on accessions is partially responsible for the lack of passion fruit breeding programs and may have also resulted in the reduced productivity of existing programs.

The vast majority of passion fruit accessions are maintained in Brazilian institutions, highlighted by the collections and germplasm banks maintained by Embrapa (Centro de Pesquisa Nacional de Mandioca e Fruticultura, and Centro de Pesquisa Agropecuário do Cerrado), the Instituto Agronômico de Campinas (IAC), and the Instituto Agronômico do Paraná (IAPAR) as well as the Universidade Estadual Paulista (UNESP), Universidade Estadual do Norte Fluminense (UENF), Universidade Federal do Rio de Janeiro (UFRJ), and Escola Superior de Agricultura Luiz de Queiroz (ESALQ/USP) [25]. The current estimate is that these eight institutions have 65 passion fruit species represented in 640 accessions, approximately 25% of which are related to the two species of greatest commercial interest, namely, *P. edulis* (with 105 accessions) and *P. alata* (with 65 accessions) [25,59].

The discrepancy between the natural variability inherent to the genus and the reduced variability represented in BAGs should be remedied as quickly as possible because it hinders the establishment and progression of *Passiflora* genetic breeding programs [59]. Similarly, utilization of the available genetic variability for *Passiflora* has been neglected during cultivar generation [61]. Thus, although several wild passion fruit species are resistant to diseases and pests, derived cultivars do not have the same genetic resistance. In fact, these cultivars are prone to contracting the major diseases of cultivated passion fruit. Furthermore, the literature suggests that different cultivars exhibit low variability in their levels of resistance [32].

The above conditions are justified, at least partially, because the first studies in the 1980s [28,71] that contributed to the establishment of *Passiflora* breeding programs prioritized production (quantitatively and qualitatively) and not necessarily the other aspects of genetic diversity, such as resistance, ornamentation, and the production of potential medicinal compounds.

In addition to the potential for commercialization because of the natural beauty of their flowers and the taste of their fruits, wild passion fruit species have desirable characteristics for the yellow passion fruit, including resistance to pathogens and pests (*P. caerulea*, *P. cincinnata*, *P. coccinea*, *Passiflora gilberti*, *P. laurifolia*, *P. setacea*, and *P. suberosa*), self-compatibility (*P. tenuifolia*, *P. elegans*, *P. capsularis*, *P. villosa*, *P. suberosa*, *P. morifolia*, and *P. foetida*), and variability in the season of production compared with commercial species (*P. setacea* and *P. coccinea*) [33,54]. For some species, such as *P. caerulea*, *P. cincinnata*, *P. coccinea*, and *P. setacea*, interspecific hybrids are being developed and characterized [33,54,72] and estimates of genetic diversity and confirmation of the hybrids based on molecular markers are being carried out [54,62,63].

According to these studies, the genetic base of the yellow passion fruit is restricted with respect to disease resistance and tolerance. In fact, the authors favor the use of genetic resources from wild species in breeding programs. Several authors have emphasized research on the description, characterization, and use of germplasm as a priority for passion fruit production to obtain more productive passion fruit cultivars with increased resistance to diseases [54,73]. The joint use of molecular markers and classical breeding procedures has been suggested as a necessary strategy to accelerate the production of fruit varieties that are adapted to different Brazilian regions [64].

Although the realities of existing cultivars, such as low variability and susceptibility to disease, still endure for cultured passion fruit, an increasing number of studies devoted to the characterization of resistance in wild species and commercial accessions are being performed [32,34,74,75]. Interspecies crosses that aim to incorporate the resistance genes of wild species into commercial species are also being implemented and evaluated [54,76,77]. The following sections discuss the applications and contributions of molecular markers to our understanding of the genetic diversity of *Passiflora* and to the various stages of breeding programs.

### 2.2. Application of Molecular Markers for the Characterization of Passiflora Diversity

The use of molecular markers for genetic characterization projects with a variety of objectives for a range of organisms is routine in many laboratories. Although molecular biology techniques have become prevalent in recent decades, leading to a reduction in the cost of most molecular markers, the availability of resources and background information are still determining factors in the choice of methods and markers to be used. In recent decades, the increase in the number of molecular markers associated with the growth of related fields of study, such as population genetics, genetic breeding, statistics, and bioinformatics, has generated immense progress in the knowledge applicable to conservation and biodiversity manipulation.

**Figure 5:**
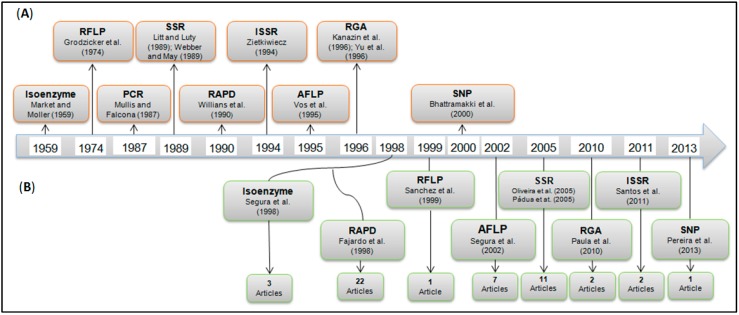
Chronological schematic illustrating the main molecular markers and their initial reference (**A**) as well as the published articles using molecular markers in the genus *Passiflora*, their initial reference, and the total number of papers (**B**).

Genetic studies associated with molecular markers are becoming standard for the *Passiflora* genus. These studies range from the use of dominant markers to the development and employment of co-dominant markers and single-nucleotide polymorphisms (SNPs). Figure 5 provides a general chronological overview of the evolution of major molecular markers and their use in studies of *Passiflora*. A simple quantitative evaluation of published papers, the data from which are shown in Figure 5, indicates that the use of molecular markers in *Passiflora* research began approximately 15 years ago with the use of the random amplified polymorphic DNA (RAPD) markers to study the diversity of 52 accessions from 14 *Passiflora* species [7].

Despite the availability of approximately 4000 fragments of DNA and RNA sequences from *Passiflora* (data from the NCBI nucleotide database [78], genetic studies employing widely used molecular markers in different stages of breeding programs and for estimates of diversity (for example, restriction fragment length polymorphism (RFLP), RAPD, amplified fragment length polymorphism (AFLP), inter-simple sequence repeat (ISSR), simple sequence repeat (SSR), and SNP markers) have typically been limited to a few *Passiflora* species. However, additional species are currently being studied. Table 2 (and the supplementary Information) provides a summary of existing research using molecular markers during different phases of genetic breeding (from pre- to post-breeding) regardless of the study’s scope. Table 2 includes all of the information about these studies, ranging from the investigation goals to the techniques and species used. The data in Table 2 confirm the absence of genetic and molecular information reflected by the genetic diversity of *Passiflora*; few species have been studied (fewer than 60), and most of the research has focused on *Passiflora* species or accessions with previously established commercial interest. Research on ecological justifications and aims coupled with species conservation have not been performed, although brief discussions on the characterization and *ex situ* conservation have been published.

Most molecular genetic studies (approximately 65% of all papers, 85% of which are based on genetic diversity) are based on the use of dominant markers. Although dominant markers are useful for genetically distinguishing species (for tests to confirm hybrids and the direction of convergent and divergent breeding), they have limitations, such as low repeatability and suboptimal genetic information profiles (homozygote and heterozygote specimens cannot be distinguished), that are highly relevant for population studies.

Despite the limitations inherent in the use of dominant markers, genetic diversity estimates based on wild and commercial accessions have contributed to the understanding of the genetic variability of passion fruit. In this context, RAPD and AFLP markers have been used to demonstrate high genetic variability among accessions of *P. edulis* [58,79] as well as a lack of correlation between the estimated genetic distances and geographical origins of the accessions evaluated [79]. However, estimates of variability using RAPD markers performed with commercial accessions of *P. edulis* indicated low genetic variability [8,80]. The authors of these studies have suggested that the apparent contradiction between the low genetic diversity identified with molecular markers and the large variability observed in commercial plantations is due to the major influence of the environment on morphological characteristics. Greater genetic distance was observed among accessions of purple and yellow fruits [8,58], and the accessions of purple and yellow fruits are considered genetically distinct despite belonging to the same species [58].

The use of RAPD markers has also contributed to estimates of diversity in wild species of passion fruit, such as *P. cincinnata*, *P. nitida*, and *P. setacea* [62,63,81]. In these studies, large genetic variability was observed, with percentages of polymorphic loci ranging from 64% in *P. nitida* [81] to 93% in *P. setacea* [63]. The fact that many studies, especially those conducted on wild species, only address accessions collected in the same geographic region illustrates the need to conduct further studies with a broader representation of accessions to more completely determine the variability and possible genetic structure of passion fruit.

Results on the characterization of genetic variability and the identification of potential regions related to resistance genes, phylogenetically conserved in plants, were first published for the genus *Passiflora* in 2010 [82] (Figure 5 and Table 2). This study used six degenerate combinations of resistance gene analogs (RGA) primers, originally designed to anneal with the NBS (nucleotide binding site) motive present in several classes of resistance genes. The results presented by Paula *et al.* [82] show wide interspecific variability among the amplicons generated, enabling the use of these markers for variability studies in germplasm banks as well as in genetic mapping. Although these are preliminary results, the authors also observed amplicons containing genomic segments of type NBS-LRR (leucine-rich protein and the nucleotide binding sites) present in different plant species.

Since the publication of these results, studies of intra-and interspecific diversity performed with use of RGA markers are being conducted at the Universidade Estadual do Sudoeste da Bahia (UESB, Itapetinga, Bahia), and the preliminary results confirm the efficiency of these markers for the characterization of genetic variability in commercial and wild species of passion fruit [83]. RGA markers have also contributed to linkage analysis and the construction of a recent genetic map for *P. alata* [84].

**Table 2:** Descriptive and quantitative presentation of papers published using different molecular markers in the genus *Passiflora*.

Molecular Markers	General Aim of the Article	Evaluated Species	No. of Species	No. of Articles
Isozymes	Estimation of diversity (germplasm)	*P. ampullacea*, *P. antioquensis*, *P. bracteosa*, *P. cumbalensis*, *P. manicata*, *P. mixta*, *P. pinnatistipula*, *P. tarminiana*, *P. tripartita*	10	3
RFLP	Estimation of diversity (germplasm)	*P. edulis*, *P. ligulares*, *P. maliformis*, *P. caerulea*, *P. mollissima*, *P. sp. india*, *P. cumbalensis*, *P. antioquiensis*, *P. pinnatistipula*, *P. x rosea*, *P. adenopoda*, *P. coriacea*	12	1
RAPD	Estimation of diversity (germplasm)	*P. adenopoda*, *P. alata*, *P. amethystina*, *P. antioquiensis*, *P. caerulea*, *P. capsularis*, *P. cincinnata*, *P. coccinea*, *P. coriacea*, *P. cumbalensis*, *P. edulis*, *P. foetida*, *P. gibertii*, *P. laurifolia*, *P. ligularis*, *P. macrocarpa*, *P. malacophylla*, *P. maliformis*, *P. micropetala*, *P. mollissima*, *P. morifolia*, *P. mucronata*, *P. nitida*, *P. palmeri*, *P. pinnatistipula*, *P. serrato*, *P. setacea*, *P. spinosa*, *P. suberosa*, *P. subpeltata*, *P. trintae*, *P. vitifolia*, *P. xrosea*	33	15
	Characterization and confirmation of hybrids	*P. alata*, *P. gardneri*, *P. gibertii*, *P. foetida*, *P. sublanceolata*, *P. watsoniana*	6	3
	Genetic mapping	*P. edulis*	1	1
	Other	*P. actinia*, *P. alata*, *P. amethystina*, *P. caerulea*, *P. coccinea*, *P. eichleriana*, *P. edulis*, *P. galbana*, *P. glandulosa*, *P. gibertii*, *P. laurifolia*, *P. mucronata*, *P. nitida*, *P. sidaefolia*, *P. setacea*	15	2
AFLP	Estimation of diversity (germplasm)	*P. alnifolia*, *P. ampullacea*, *P. antioquensis*, *P. bracteosa*, *P. cumbalensis*, *P. edulis*, *P. fimbratistipula*, *P. gracilens*, *P. ligularis*, *P. mixta*, *P. manicata*, *P. parritae*, *P. pinnatistipula*, *P. popenovii*, *P. tarminiana*, *P. tenerifensis*, *P. tiliaefolia*, *P. tripartita*, *P. trifoliata*, *P. trinervia*	20	3
	Genetic mapping	*P. alata*, *P. edulis*	2	2
SSR	Estimation of diversity (germplasm)	*P. capsularis*, *P. edulis*, *P. rubra*	2	4
	Characterization and confirmation of hybrids	*P. foetida*, *P. sublanceolata*	2	1
	Genetic mapping	*P. edulis*	1	1
	Development, characterization and selection of markers	*P. alata*, *P. cincinnata*, *Passiflora contracta*, *P. edulis*, *P. setacea*	5	7
	Cross-amplification	*P. caerulea*, *P. cincinnata*, *P. edulis*, *P. foetida*, *P. gibertii*, *P. ligularis*, *P. maliformis*, *P. mucronata*, *P. rubra*, *P. setacea*, *P. suberosa*	11	2
	Other	*P. alata*	1	1
RGA	Estimation of diversity (germplasm)	*P. caerulea*, *P. coccinea*, *P. edulis*, *P. gibertii*, *P. nitida*, *P. odontophyla*, *P. serratodigitata*, *P. setacea*	8	1
	Genetic mapping	*P. alata*	1	1
ISSR	Estimation of diversity (germplasm)	*P. alata*, *P. edulis*	2	2
SNP	Genetic mapping	*P. alata*	1	1
Other markers	Estimation of diversity (germplasm)	*P. edulis*	1	1
	Genetic mapping	*P. alata*	1	1

RFLP, restriction fragment length polymorphism; RAPD, random amplified polymorphic DNA; AFLP, amplified fragment length polymorphism; SSR, simple sequence repeat; RGA, resistance gene analogs; ISSR, inter-simple sequence repeat; SNP, single-nucleotide polymorphism; The data presented were obtained from queries of the Scopus [69] and Web of Science [70] databases. Detailed information on the references used to construct this table is available in the supplementary material.

Studies on *Passiflora* using co-dominant markers began in 2005 and were mainly limited to the development of microsatellite markers for the species *P. edulis* [85,86,87], *P. alata* [88,89], *P. cincinnata* [87,90], *P. setacea* [87], and *P. contracta* [91]. Studies on other species of this genus addressed the evaluation of cross-amplification [15,92,93,94] and hybrid confirmation [45]. Most of these studies were restricted to characterizations in agar gels or polyacrylamide gels and require additional evaluation, including profiles of amplifications and polymorphic loci. The identification and characterization of microsatellite loci in the genus *Passiflora* indicated low levels of polymorphism in these genomic regions, at least for the species evaluated (Table 3). The average number of alleles per characterized microsatellite locus was low in studies of wild and commercial species of *Passiflora*, ranging from 2.8–5 alleles per locus for the wild species *P. cincinnata*, *P. contracta*, *and P. setacea* [87,90,91] and from 3.1–7.6 alleles per locus for the species of greatest commercial interest, namely, *P. edulis* and *P. alata* [85,88].

Regarding the estimates observed for microsatellite loci from passion fruit (Table 3), it is also important to note that the low values of observed heterozygosity, compared with the values of expected heterozygosity, suggest low allelic diversity and tendency toward the fixation of alleles, at least for the germplasm evaluated. The percentage of polymorphic microsatellite loci observed in characterizations of passion fruit species can also be considered low, with an average of approximately 28% of microsatellite loci polymorphic across wild and commercial species; in the wild species *P. cincinnata* and *P. setacea*, the individual values are 21% and 29%, respectively [86,89], and in the commercial species *P. alata* and *P. edulis*, the individual values are 25% and 35%, respectively [86,89].

**Table 3:** Average values observed for the number of alleles (*Na*) and observed (*H*_0_) and expected (*H*_E_) heterozygosity in the characterization of microsatellite loci from passion fruit species (*Passiflora* spp.).

Species	Characteristics of Microsatellite Loci			References
	*Na*	*H*_0_	*H*_E_	
*P. alata*	3.1	0.26	0.53	Pádua *et al.* [82]
*P. cincinnata*	5	0.52	0.52	Cerqueira-Silva *et al.* [84]
*P. cincinnata*	3.3	0.26	0.36	Cerqueira-Silva *et al.* [81]
*P. contracta*	4.9	0.53	0.61	Cazé *et al.* [85]
*P. edulis*	7.6	0.58	0.62	Oliveira *et al.* [79]
*P. edulis*	3.4	0.31	0.36	Cerqueira-Silva *et al.* [81]
*P. setacea*	2.8	0.34	0.41	Cerqueira-Silva *et al.* [81]

Research that characterizes genetic diversity in wild populations by microsatellite markers remains unpublished, although reports on evaluations of germplasm materials in Colombia [95] and Brazil [94,95,96,97,98] have been published. The microsatellite marker sets used by Ortiz *et al*. [95] in a study with *P. edulis* and by Amorim *et al*. [96] in a study with *P. capsularis* and *P. rubra* have not shown polymorphisms, whereas other studies performed by Reis *et al.* [97,98] have exclusively evaluated genotypes from cycles of recurring selection in *P. edulis* progenies. The hypothesis that microsatellite loci have low levels of polymorphism, which has been based on identification and characterization studies of the microsatellites [85,86,87,88,89,90], is supported by the absence of polymorphism observed in a study of genetic variability in Colombia that analyzed 70 passion fruit plants (*P. edulis*) collected in 60 commercial plantations [95]. Ten microsatellite loci reported in *P. edulis* [85] and seven in *P. alata* [88] were used in that study.

The first successful estimates of genetic diversity in *Passiflora* accessions were obtained with SSR markers in 2013 [92,94]. However, those studies were limited in their estimates of intraspecies diversity because of the small number of plants that were evaluated (fewer than six plants for most species). In addition, even for interspecific studies, such as that performed by Oliveira *et al.* [92], where 11 passion fruit species were considered, the average number of alleles was not more than five (ranging from 1–13 alleles per locus).

The variation in the genetic diversity estimated in published papers, primarily those analyzing commercial species, is due to not only the specific characteristics of the genotypes evaluated but also the effects of the different statistical methodologies employed by the authors. Methodological influences on the results of diversity estimates have been reported for a variety of species, including *Mangifera indica* [99], *Olea europaea* [100], *Zea mays* L. [101], and *Solanum lycopersicum* [102]. Differences in methodologies have also been discussed for studies of morphological characteristics and of genetic diversity that used molecular markers to study passion fruit [103]. In the latter work involving passion fruit, comparisons were made among seven distance measures, 14 similarity coefficients, and five grouping methods. The authors concluded that the diversity estimate is significantly influenced by the method employed. Considering the different methodological strategies that have been employed for data analysis in the various studies of diversity among passion fruit accessions, the authors suggest that the choice of the methodology to be used and the comparison of results obtained using different measurements and coefficients should be performed carefully.

Research projects conducted as part of a joint venture between the Universidade Estadual de São Paulo (ESALQ/USP, Piracicaba, Brazil) and Centre National de Ressources Génomiques Végétales (CNRGV/INRA, Toulouse, France) during the past five years have introduced the first SNP identifications in commercial passion fruit species (*P. alata*) [84]. However, the use of these data remains limited to the construction and saturation of genetic maps, as is the case for most microsatellites available for commercial *Passiflora* species.

### 2.3. Application and Perspectives of Genetic Engineering for Passion Fruit Culture

Considering the diversity of species and intra- and interspecific genetic variability available for use in breeding programs of passion fruit, genetic engineering as a methodological strategy to increase favorable characteristics for passion fruit culture has been specifically employed to search for disease resistance [104]. With regard to diseases such as passion fruit woodiness (PWD) (caused in Brazil by *Cowpea aphid-borne mosaic virus*) and the bacterial spot disease (caused by *Xanthomonas axonopodis* pv. *passiflorae*), for the which chemical control measures are not effective and resistant cultivars are not available, a growing number of advances in the use of genetic engineering have been observed in recent decades [105,106,107,108].

Studies attempting to produce transgenic passion fruit have been conducted since the late 1990s and early 2000s at the Universidade Federal de Viçosa (UFV), Minas Gerais, Brazil, and Universidade de São Paulo (USP), São Paulo, Brazil; the first results were published in 2005 [105] and 2006 [106]. In these two studies, plants of yellow passion fruit (*P. edulis*) were transformed from fragments of genes obtained from viral isolates known to cause PWD. These studies were initiated based on a pathogen-derived resistance (PDR) approach that likely relied on the induction of post-transcriptional gene silencing (PTGS).

An untranslatable construct consisting of two-thirds of the *NIb* gene and the 5' region of the CP gene derived from a viral isolate of CABMV was used by Alfenas *et al.* [105]. In turn, a construct consisting primarily of the *CP* gene was used by Trevisan *et al.* [106]. In both studies, transgenic plants resistant to PWD were obtained. However, the effective resistance observed in the transformed plants by Alfenas [105] was restricted to the viral isolate from which the fragments of the *NIb* and *CP* genes were isolated. It is probable that the high variability in the nucleotide sequence of the *NIb* gene was the main factor contributing to this result. Subsequently, the transgenic plants (R_0_) were self-crossed to generate homozygous lines (R_1_), and a plant of this line was resistant to seven viral isolates [109]. The authors defend the hypothesis that the dose used, due to the presence of homozygous plants for the transgene in the R_1_ lineage, contributes substantially to the spectrum of resistance observed [105,109].

In addition, one of the transgenic plants produced by Trevisan *et al.* [106] was immune to all the three isolates considered in your search, but the potential resistance of these transgenic lines to other isolates found in Brazil is not known. Studies performed by Monteiro-Hara [107] identified new transgenic lines of passion fruit and generated transgenic lines R_1_ and R_2_, both from transgenic plants characterized by Trevisan *et al*. [106], as well as from the new lines constructed. Homozygous lines did not show symptoms of the viral disease after either mechanical inoculation or inoculation by aphids [107]. New crosses were made between R_2_ plants selected by the authors for future studies.

Although in smaller proportions or in less advanced stages, studies devoted to obtaining transgenic plants were also conducted with *P. edulis* for resistance to diseases and herbicides through the introduction of a baculovirus anti-apoptotic gene (*p35* gene) [108] and the resistance to bacterial spot disease by introducing the sequence of the attacin A gene (*att*A) [110]. Plants of *P. edulis* transformed with *p35* gene sequences showed no resistance to CABMV but showed increased tolerance to bacterial spot disease and glufosinate herbicide when compared to non-transgenic plants [108]. Ten plants of *P. edulis* transformed with the *att*A sequence of the gene showed no leaf lesions after inoculation with *X. axonopodis* pv. *Passiflorae*, indicating possible resistance to the pathogen. Initial tests attempting to obtain transgenic plants were conducted with *P. alata* [111]. These experiments demonstrated the possibility of obtaining transgenic plants for *P. alata*. Similar to the work conducted with *P. edulis*, the transformations achieved in *P. alata* were conducted using *Agrobacterium tumefaciens* and a fragment of the *CP* gene CABMV.

In conjunction, the progress observed with the studies dedicated to the production of transgenic passion fruits indicates that, at least for PWD, it is possible that resistant plants will soon become available for cultivation and/or for use in breeding programs dedicated to the production of resistant cultivars with higher productivity.

### 2.4. Contributions and Perspectives of Molecular Biology in Pre- and Post-Breeding Programs

The contributions of molecular biology, and especially the use of the molecular markers, in (pre-) breeding programs may be didactically separated into at least six research modes: (i) the direction of convergent and divergent crossings leading the initial stages of breeding; (ii) the confirmation of intra- and interspecies hybrids with a reduction in the occurrence of escapes; (iii) the contribution of retro-crossings with a decrease in the time necessary for the re-composition of the genome; (iv) the genetic mapping and identification of quantitative trait loci (QTLs) that are useful for the subsequent introgression of genes into commercial species and cultivated varieties; (v) the prospection and characterization of resistance genes, both by mapping strategies and through the use of analog markers of resistance genes; and (vi) the identification and protection of hybrids and cultivars. Coupled with the effective use of markers, these joint activities contribute to the establishment of an assisted selection program because all of the information provided enables the efficient use of the available germplasm.

In addition to their contribution to understanding the diversity of populations or groups of regional accessions, estimates of passion fruit genetic diversity have enabled the identification of converging and diverging crosses that can contribute to different stages of a (pre-) breeding program. For commercial species, such as *P. edulis* [80], as well as wild species, such as *P. setacea* [63] and *P. cincinnata* [62], preferred crossings were suggested after the characterization of RAPD markers to generate progenies with greater or lower potential for segregating characteristics. The identification of genetically divergent accessions between yellow and purple passion fruit that has been achieved based on molecular markers [8,58] can also be used to target crosses that aim to broaden the genetic base of breeding programs for *P. edulis*.

The absence of agronomic characterizations for most accessions in the aforementioned studies is the greatest challenge to introducing information from genetic diversity estimates into current breeding programs. Difficulties in the joint use of data from phenotype and genotype characterizations (particularly when related to neutral markers) have been reported for different cultures and are an aspect of the challenge inherent in studies of natural populations [112]. For cultivar development, the pre-breeding programs that completely exploit the use of molecular techniques and field characterizations in germplasm are indicated as bonding strategies between natural variability and the cultivars employed for the maintenance and growth of cultures [67].

Different molecular markers, such as RAPD and microsatellites, are highly efficient and user-friendly techniques that can be used to confirm intra- and interspecies hybrids. The first study to employ molecular markers to confirm hybrids from 17 interspecies crosses of 14 *Passiflora* species of agronomic interest associated with fruit production was published in 2008 [72] and was based on the profile of DNA fragments generated using 12 RAPD primers. Similar molecular markers have since been utilized to confirm and characterize interspecific ornamental hybrids [47,113] based on the profile of DNA fragments generated using seven RAPD primers and one SSR primer. The confirmation of hybrids using molecular markers may decrease the occurrence of escapes and reduce costs via the restricted maintenance of seedlings to be used in sequential stages of a (pre-) breeding program.

Molecular markers also contribute to the characterization and selection of specimens with a higher genomic contribution from the recurrent parent [76,114]. The characterization of parents and segregant genetic diversity, which are associated with evaluations of the specific phenotype, inform crossings and reduce the number of backcrossing cycles required to obtain the hybrid of interest at the start of breeding. This strategy has improved the introgression of disease resistance found in the wild species *P. setacea* into the cultivars GA-2, AR-1, and EC2-O of *P. edulis* [ 114]. An experiment performed at EMBRAPA Cerrados re-established a 92% average of the commercial ancestor’s genome (*P. edulis*) in hybrids while maintaining the resistance of the wild ancestor (*P. setacea*) [114].

One of the most thorough passion fruit studies, at least with regard to molecular markers within the context of breeding, consists of the construction of genetic maps and identification of regions associated with characteristics of interest (QTLs or, more specifically, quantitative resistance loci, QRLs). Early genetic mapping of passion fruit species began in 2000, and the first maps were published in 2002 [115]. These maps were based on the characterization of a segregant population of *P. edulis* using RAPD markers. The population used to construct the first map helped to identify the first QRLs linked to the genus *Passiflora* using AFLP markers [116]. The quantitative resistance locus detected by Lopes *et al.* [116] explained approximately 16% of the phenotypic variation related to symptoms from bacterial spot disease caused by *X. axonopodis* pv. *passiflorae*.

The use of map integration strategies to construct a single map (rather than two maps representing ascendants) [86] was a landmark in genetic mapping studies and the identification of QTLs in passion fruit. Another important feature of the *Passiflora* map was the use of microsatellite markers for the map construction in addition to AFLP [86]. An important advantage of the integrated construction of a single representative map of a segregant population is an increase in map saturation and length. The extension of research to construct maps and the identification of QTLs in passion fruit plants have occurred only recently for another species of this genus: the first integrated genetic map for *P. alata* was published in 2013 [104]. The use of variable AFLP and SSR techniques and the first characterization of SNPs in the passion fruit plant were included in this map [104].

Research dedicated to the construction of physical maps has also been conducted [42]. Physical maps were obtained from putative genes identified from a passion fruit genomic library inserted into BACs [42,43]. This research is a new development in genetic and genomic studies of passion fruit; it characterizes the species’ genomes, identifies evolutionary relationships, and develops new molecular markers, including SSR markers, expressed sequence tags (ESTs), and SNPs.

The advances in the genetic mapping of passion fruit associated with the reduction of costs of next-generation sequencing (NGS) should enable genetic characterizations based on genotyping-by-sequencing (GBS), at least for commercial species, in both the short and medium term. In this context, alternative methodologies that reduce costs and enable GBS, such as the use of restriction enzymes to reduce genome complexity [117], should be considered in future genetic studies of passion fruit; these methodologies can contribute to both population studies and procedures of genome-wide selection in collections and germplasm banks.

Molecular markers are also used in different stages: after cultivars are obtained and distributed (generically termed post-breeding), when paternity is determined (and thus the culture is protected), and when monitoring cultivar genetic purity, regardless of whether the cultivars are clonal or seminal [64]. When legislation to protect cultivars is in effect (Act 9456, Brazil), the growth, development, and use of marker groups for post-breeding will be increasingly necessary for passion fruit cultivation, particularly because of the increase in the number of varieties that will be developed and made available to producers.

## 3. Conclusions, Perspectives, and Challenges

Considering the challenges of breeding passion fruit, as well as the available knowledge and conservation of the genetic variability of this genus, we believe that the following research activities should be prioritized: (i) the identification of new accessions to increase the representative breadth of germplasm banks, especially for wild species; (ii) the measurement of population genetics estimates that enable a true understanding of the genetic diversity and structure of passion fruit; (iii) phenotypic characterizations that contribute to both the useful evaluations of the germplasm for interspecific crosses and studies of the association between phenotypic and molecular data; (iv) research efforts devoted to identifying genomic regions associated with resistance to diseases; and (v) the potential use of transgenic strains previously evaluated in crosses targeted in breeding programs.

The recent research on the diversity of wild passion fruit species and the increasing use of molecular markers, particularly co-dominant markers, have made knowledge from population studies available and are promising for further genetic research on passion fruit. Although highly relevant for conservation and breeding activities, population studies of *Passiflora* are only in the initial stages. Coupled with a large gap in group population genetic research, the increasing availability of microsatellite markers for *Passiflora* species and the use of cross-amplification strategies to popularize marker use should increase the number of research studies. Further research should be performed to identify and characterize microsatellite loci because the current results indicate low allelic diversity among SSR loci. In this context, the use of strategies for sequencing and genotyping on a large scale will be important.

In parallel with the population estimates that must be performed for the genus *Passiflora* via molecular markers, strategies for the exploration and conservation of germplasm collections are also necessary to enable morpho-agronomic characterizations to be performed efficiently to increase the understanding of both neutral biological diversity and diversity that is subject to natural selection. In this way, the interactions involving the accessions present in banks, germplasm collections, and elite materials used in breeding programs will be enhanced.

Similar to the expected growth in microsatellite marker usage in population studies, recent genomic investigations of commercial passion fruit species have developed and characterized SNP markers. These studies should be promoted because they will encourage different types of research, as has occurred with the saturation of genetic maps, the identification of QTLs, and the exploitation of mapping by association. Collections, germplasms, or elite accessions can be employed in such a strategy, and thus, a detailed map of the genomic regions of interest can be achieved. Moreover, the development and use of large-scale genotyping should allow for the use of strategies for genome-wide selection (or simply genomic selection), enhancing the association of genetic diversity data with characteristics of agronomic interest. In addition, the use of GBS strategies should help to reduce the costs and time required for germplasm characterization and the selection of passion fruit genotypes.

## Supplementary Information

**Table S1:** Descriptive and quantitative presentation of papers using molecular markers in the genus *Passiflora*.

Molecular Markers	General Aim of the Article	No. of Article	References
Isoenzyme	Estimation of diversity (germplasm)	3	[118,119,120]
RFLP	Estimation of diversity (germplasm)	1	[121]
RAPD	Estimation of diversity (germplasm)	15	[7,8,58,62,63,80,81,122,123,124,125,126,127,128,129]
	Characterization and confirmation of hybrids	3	[47,72,113]
	Genetic mapping	1	[115]
	Others	2	[114,130]
AFLP	Estimation of diversity (germplasm)	3	[79,95,131]
	Genetic mapping	2	[84,86]
SSR	Estimation of diversity (germplasm)	4	[95,96,97,98]
	Characterization and confirmation of hybrids	1	[47]
	Genetic mapping	1	[86]
	Development, characterization and selection of markers	7	[85,86,87,88,89,90,91]
	Cross-amplification	2	[87,92]
	Others	1	[130]
RGA	Estimation of diversity (germplasm)	1	[82]
	Genetic mapping	1	[84]
ISSR	Estimation of diversity (germplasm)	2	[132,133]
SNP	Genetic mapping	1	[84]
RGA	Estimation of diversity (germplasm)	1	[134]
	Genetic mapping	1	[84]

RFLP, restriction fragment length polymorphism; RAPD, random amplified polymorphic DNA; AFLP, amplified fragment length polymorphism; SSR, simple sequence repeat; RGA, resistance gene analogs; ISSR, inter-simple sequence repeat; SNP, single-nucleotide polymorphism.
